# Overexpressed lncRNA ROR Promotes the Biological Characteristics of ox-LDL-Induced HUVECs *via* the let-7b-5p/HOXA1 Axis in Atherosclerosis

**DOI:** 10.3389/fcvm.2021.659769

**Published:** 2021-09-13

**Authors:** Cong Yu, Bin Wu, Jinsong Jiang, Guangwei Yang, Chao Weng, Fei Cai

**Affiliations:** ^1^Department of Vascular Surgery, Vascular Interventional Center, Zhejiang Provincial People's Hospital, People's Hospital of Hangzhou Medical College, Hangzhou, China; ^2^Department of Surgery, Pinghu Traditional Chinese Medicine Hospital, Pinghu, China; ^3^Department of Vascular Surgery, Wuhan Union Hospital, Tongji Medical College, Huazhong University of Science and Technology, Wuhan, China

**Keywords:** atherosclerosis, oxidized low-density lipoprotein, regulator of reprogramming, let-7b-5p, Homeobox A1

## Abstract

The long non-coding RNA regulator of reprogramming (lncRNA ROR) is involved in atherosclerosis (AS), but the specific mechanism remains unclear. The expressions of lncRNA ROR, let-7b-5p, Homeobox A1 (HOXA1), and apoptosis-associated proteins in the serum of AS patients and human umbilical vein endothelial cells (HUVECs) were determined by quantitative real-time PCR (qRT-PCR) and Western blot. The relationships of lncRNA ROR, let-7b-5p, and HOXA1 were analyzed by Pearson's correlation test. The viability and the migration of HUVECs were measured by Cell Counting Kit-8, wound healing, and Transwell assays. The predicted target gene and the potential binding sites were confirmed by dual-luciferase reporter assay. lncRNA ROR was highly expressed in AS, which promoted the cell viability and migration of HUVECs, while lncRNA ROR silencing produced the opposite results. The expression of let-7b-5p, which bound to lncRNA ROR, was downregulated in AS, indicating that the two genes were negatively correlated. Besides this, let-7b-5p reversed the effects of upregulated lncRNA ROR expression on let-7b-5p expression, cell viability, and migration as well as the expressions of apoptosis-related proteins of ox-LDL-treated HUVECs. HOXA1 was targeted by let-7b-5p and upregulated in AS, with its expression being negatively correlated with let-7b-5p but positively correlated with lncRNA ROR. In ox-LDL-treated HUVECs, overexpressed HOXA1 reversed the effects of let-7b-5p, and HOXA1 silencing reversed the effects of lncRNA ROR. In AS, lncRNA ROR promoted the biological characteristics of oxidation of low-density lipoprotein-induced HUVECs *via* the let-7b-5p/HOXA1 axis.

## Introduction

Atherosclerosis (AS), characterized by the continuous accumulation of lipids and inflammatory cells in the intima of large arteries, is a chronic cardiovascular disease and one of the most common causes of death among the elderly (1, 2). Nowadays, as most heart attacks and strokes are mainly caused by the unstable plaque rupture of AS, some anti-thrombotic, statins, and anti-hypertensive drugs have been developed to reduce these adverse events; however, they could only delay the progression of AS (3). Studying the pathogenesis of AS from the perspective of its molecular mechanisms in AS could also contribute to a comprehensive understanding of AS. Endothelial dysfunction, as one of the main risk factors for AS, is also considered as a marker of early atherosclerosis (4). The apoptosis of endothelial cells induces lipid accumulation, monocyte adhesion, and inflammatory response, leading to the occurrence of AS (5). In addition, the abnormal proliferation and migration of endothelial cells will also trigger the occurrence of AS (6). Thus, finding the regulatory mechanism of endothelial function in AS may make a profound impact upon the AS treatment.

Long non-coding RNAs (lncRNAs), possessing more than 200 nt in total length without a protein-encoding ability, are widely involved in human diseases (7). Recently, lncRNA regulator of reprogramming (ROR) has been found to be implicated in some human diseases, including cardiovascular disease (8). lncRNA ROR, whose expression was found to be upregulated in patients with ischemia/reperfusion (IR) and in hypoxia/reoxygenation-treated myocardial cells, aggravated I/R injury by suppressing cell viability and promoting apoptosis (9). Moreover, lncRNA ROR regulated the IR injury-induced inflammatory response in human cardiac myocytes by regulating the miR-124-3p/TRAF6 axis (10) and sponged miR-138 to aggravate hypoxia/reoxygenation-induced cardiomyocyte apoptosis *via* upregulating Mst1 (11). In addition, promoting lncRNA ROR expression in homocysteine-induced aortic smooth muscle cells enhanced the progression of AS *via* the microRNA (miR)-195-5p/fibroblast growth factor 2 (FGF2) axis (12). However, the interaction between lncRNA ROR and let-7b-5p in AS remained to be comprehensively elucidated, which therefore is the focus of the present study.

lncRNAs sponge miRNAs to affect the expressions or functions of miRNAs (13). miRNAs (19–25 nt in length), as a family of small and highly conserved ncRNAs, regulate gene expressions through binding to their 3′-untranslated regions (3′-UTRs) (14). let-7b has a protective effect on the regulation of inflammation in diabetes-related AS (15). It has been reported that the failing heart released let-7b-5p (16) that could competitively bind to lncRNA small nuclear RNA host gene 16, facilitating G2/M cell cycle transition and epithelial-to-mesenchymal transition *via* the let-7b-5p/CDC25B/CDK1 axis (17). The downregulation of let-7b exerted a proatherosclerotic effect through enhancing endothelial apoptosis *via* targeting HAS-2 and suppressing the activation of the P13k/Akt signaling pathway (18). However, the biological role of let-7b-5p and its interaction with lncRNA ROR in AS were hardly discussed. The oxidation of low-density lipoprotein (ox-LDL) has been found to promote AS (19). ox-LDL plays a central role in atherosclerosis by acting on multiple cells through lectin-like oxidized LDL receptor-1 (LOX-1), and naturally occurring compounds have been shown to modulate LOX-1 expression and AS (19–21). Our study aimed to discover the roles and functions of lncRNA ROR and let-7b-5p in ox-LDL-stimulated human umbilical vein endothelial cells (HUVECs), with the hope of developing a potential therapeutic strategy for the prevention and treatment of AS.

## Materials and Methods

### Ethical Statement

The study was conducted after obtaining an approval from the Ethics Committee of Zhejiang Provincial People's Hospital (approval number: ZPPH2018020601). All the recruited patients have signed an informed consent and agreed to the use of their tissues for clinical research.

### Clinical Specimens

Specimens of peripheral venous blood were collected from patients with AS (*n* = 30), as diagnosed by clinical symptoms and coronary angiography, as well as from healthy donors (*n* = 22) at Zhejiang Provincial People's Hospital between 2018 February and 2019 March. The clinical parameters of the AS patients and the healthy donors are shown in Table 1. The serum was separated from the blood samples by low-speed centrifugation and stored at −80°C for subsequent studies.

**Table 1:** Clinical parameters of athersclerosis patients.

**Parameters**	**Normal controls**	**Atherosclerosis patients**
Number	22	30
Sex (female,%)	36.3	23.3
Age	59.12± 6.93	62.51 ± 12.3
Total cholesterol (mmol/L)	3.83 ± 0.56	5.29 ± 0.50^*^
LDL-C (mmol/L)	2.21 ± 0.22	2.36 ± 0.35
HDL-C (mmol/L)	1.61 ± 0.25	1.70 ± 0.42
TG (mmol/L)	1.39 ± 0.15	1.97 ± 0.29^*^
Creatinine (mg/dL)	0.99 ± 0.16	1.10 ± 0.26

*Mean ± S.D.*,

* *p < 0.05*.

### Cell Culture and Transfection

The HUVECs (cat: PCS-100-010) were purchased from the American Type Culture Collection (USA) and grown in endothelial growth medium-2 (EGM-2, CC-3162, Lonza, Basel, Switzerland) supplemented with 0.5% fetal bovine serum (FBS; 16140-071, Gibco, Waltham, MA, USA) at 37°C with 5% CO_2_.

The ox-LDL (H7950) was obtained from Solarbio (Beijing, China), while small interfering RNAs for lncRNA ROR (siROR, siG150324140815-1-5) and for Homeobox A1 (siHOXA1, SR302178) were, respectively, acquired from RiboBio (Guangzhou, China) and Origene (Rockville, MD, USA). The pcDNA3.1 plasmid (V87020) was obtained from Invitrogen (Carlsbad, CA, USA), and it upregulated the lncRNA ROR and HOXA1 expressions. The let-7b-5p mimic (M; B02003) and its control (mimic control, MC; B04001) were ordered from GenePharma (Shanghai, China). The HUVECs were seeded into 24-well plates at a density of 2 × 10^5^ cells/well and grown to 80% confluence. Lipofectamine 2,000 kit (cat: 11668-019; Invitrogen, Carlsbad, CA, USA) was used for cell transfection. The transfected nucleotide sequences are listed in Table 2. After 48 h of incubation at 37°C, the cells were collected for subsequent studies.

**Table 2:** Sequences for transfection.

**Gene**	**Sequence**
siROR	5′-UAGAGCGAACAAAAAGUAGAG-3′
siHOXA1	5′-UAAGUAUGGGGUAUUCCAGGA-3′
siNC	5′-UGGUGGUCCAAAAUAUGGUGA-3′
let-7b-5p mimic	5′-UGAGGUAGUAGGUUGUGUGGUU-3′
let-7b-5p mimic control	5′-UGUGAUAGUGUGGGUUGUGAGU-3′

### Cell Viability Detection

The transfected HUVECs (5 × 10^4^ cells/well) were cultured in 96-well plates containing EGM-2 medium with 0.5% FBS at 37°C in 5% CO_2_. Then, 10 μl of cell counting kit-8 (CCK-8, C0037, Beyotime, Shanghai, China) without serum medium was added into each well to detect cell viability at 12, 24, and 36 h. The absorption value at 450 nm was measured by a microplate reader (Model 680, Bio-Rad, USA).

### Wound Healing Assay

The HUVECs (1 × 10^5^ cell/well) were grown in 24-well plates 48 h after transfection, and a wound was produced with a 1,000-μl pipette tip after the cells reached almost 100% confluence. Then, the cells were continued to culture in serum-free EGM-2 medium at 37°C. Images of the artificial wound at 0 and 36 h were visualized under an automated fluorescence microscope (BX63, Olympus, Tokyo, Japan) at × 100 magnification. Cell migration was determined by Image-Pro Plus Analysis Software 7.0 (Media Cybernetics, Rockville, MD, USA).

### Transwell Assay

Transwell chambers (8-μm pore, Corning, Inc., Corning, NY, USA) were inserted into 24-well plates. The transfected HUVECs were transferred to the upper chamber and incubated at 37°C with 5% CO_2_, whereas 600 μl EGM-2 medium with 0.5% FBS was added into the lower chamber. After incubation for 24 h, phosphate-buffered saline-rinsed cotton was used to remove the remaining cells on the upper Transwell chamber. Then, the migrated cells were treated with 4% paraformaldehyde solution for 1 h and dyed using 0.1% crystal violet for 30 min at room temperature. After washing the cells three times, the number of HUVECs was calculated from five randomly chosen fields under an automated fluorescence microscope (BX63, Olympus, Tokyo, Japan) and photographed at × 100 magnification.

### Quantitative Real-Time PCR

Total RNA from serums and cells was extracted using Trizell (15596-018, Invitrogen, Carlsbad, CA, USA) and preserved at −80°C. cDNA was synthetized from 1 μg of RNA by cDNA Synthesis Kit (K1631, Thermo Fisher Scientific, USA). The PCR reaction was performed with One-step PrimeScript RT-PCR kit (RR064B, Takara, Shiga, Japan) in a PCR Detection system (CFX96, Bio-Rad, Hercules, CA, USA) under the following conditions: at 95°C for 5 min, 40 cycles at 95°C for 30 s, at 62°C for 45 s, at 72°Cfor 90 s, and at 72°C for10 min. β-Actin and U6 were the internal controls. The sequences of the primers are listed in Table 3. The 2^−Δ*ΔCT*^ method was used for analyzing the relative expression (22).

**Table 3:** Primers for qRT-PCR.

**Gene**	**Primers**
ROR	
Forward	5′-CTCCAGCTATGCAGACCACTC-3′
Reverse	5′-GTGACGCCTGACCTGTTGAC-3′
let-7b-5p	
Forward	5′-TGGTTGTCGTATCCAGTGCAA-3′
Reverse	5′-GTATCCAGTGCGTGTCGTGG-3′
Bcl-2	
Forward	5′-GATGACTGAGTACCTGAACC-3′
Reverse	5′-AGCAGAGTCTTCAGAGACAG-3′
Bax	
Forward	5′-GACGAACTGGACAGTAACAT-3′
Reverse	5′-CTTCTTCCAGATGGTGAGT-3′
HOXA1	
Forward	5′-ACTCTGGAAATCTCTCATCTC-3′
Reverse	5′-GACTTTCATCCAGTCAAAAG-3′
U6	
Forward	5′-CTCGCTTCGGCAGCACA-3′
Reverse	5′-AACGCTTCACGAATTTGCGT-3′
β-actin	
Forward	5′-ATTGGCAATGAGCGGTTC-3′
Reverse	5′-GGATGCCACAGGACTCCA-3′

### Protein Expression Detection

Protein expressions were measured by Western blot following a previous study (23). The HUVECs were lysed by RIPA Lysis Buffer (P0013C, Beyotime, China), the proteins were collected by centrifugation, and the concentrations were quantified using a bicinchoninic acid protein kit (P0009; Beyotime, China). Next, the protein lysates (30 μg in each lane) were electrophoresed by sodium dodecyl sulfate–polyacrylamide gel electrophoresis and then transferred onto a polyvinylidene fluoride membrane (FFP32, Beyotime, China). The membrane was further blocked by 5% skimmed milk for 2 h at room temperature. After that, the membrane was incubated with the following primary antibodies: anti-HOXA1 (1:1,000, ab230513, Abcam, Cambridge, UK), anti-B-cell lymphoma-2 (Bcl-2) (1:1,000, ab32124, Abcam, UK), anti-Bcl-2 associated X protein (Bax) (1:2,000, ab32503, Abcam, UK), anti-cleaved caspase-3 (1:500, ab2302, Abcam, UK), and anti-β-actin antibody (1:10,000, ab8226, Abcam, UK) at 4°C overnight. β-Actin was the internal reference. Then, the secondary horseradish peroxidase-conjugated antibodies Goat Anti-rabbit (1:10,000, ab205718, Abcam, UK) and Goat Anti-mouse (1:10,000, ab205719, Abcam, UK) were used to further incubate the membrane at room temperature for 1 h. Finally, the gray values of the strips were analyzed by ImageJ 5.0 (NIH, Bethesda, MD, USA) and calculated with iBright CL750 Imaging System (A44116, Thermo Fisher Scientific).

### Dual-Luciferase Reporter Assay

The interactions of lncRNA ROR, let-7b-5p, and HOXA1 were predicted with RNA22 v2 microRNA target detection (http://cm.jefferson.edu/rna22/) and TargetScan (http://www.targetscan.org/vert_72/), the results of which were confirmed by dual-luciferase reporter assay. The DNA fragments of lncRNA ROR and HOXA1 containing let-7b-5p target sites (wild type or mutant) were purchased from GenePharma (Shanghai, China) and recombined with the luciferase vector (pMirGLO, cat: AM5795; Thermo Fisher Scientific, USA).

For the dual-luciferase reporter assay, the HUVECs were cultured in a 96-well plate at a density of 5 × 10^3^ cells/well and transfected with let-7b-5p M or MC and wild-type (WT) or mutated (MUT) reporter plasmids of lncRNA ROR and HOXA1 (ROR-WT, sequence: 5′-GAAAGAUCCACCUACAACCUCA-3′, ROR-MUT, sequence: 5′-GAAAGAUCCACCUACACAAGAG-3′, HOXA1-WT, sequence: 5′-CUUCCCCGGGGUCUUCUACCUCA-3′; HOXA1-MUT, sequence: 5′-CUUCCCCGGGGUCUUAGGUUGAC-3′) using Lipofectamine 2,000 reagent (Thermo Fisher Scientific, USA). After 48 h, the cells were harvested for luciferase detection, and the activity was measured using the luciferase reporter assay system (E1910; Promega, Madison, MI, USA). *Renilla* luciferase activity was calculated for the normalization of firefly luciferase activity.

### Statistical Analysis

All the experiments were independently conducted in triplicate. The data were expressed as mean ± standard deviation (SD). Statistical analysis was conducted with SPSS 20.0 software (SPSS Inc., Chicago, IL, USA). Statistical significance was assessed by paired *t*-test and one-way ANOVA, followed by Tukey's *post-hoc* test. A correlation analysis of lncRNA ROR, let-7b-5p, and HOXA1 was performed using Pearson's correlation test. Statistical significance was defined as *P* < 0.05.

## Results

### lncRNA ROR Expression Was Upregulated in AS

**Figure 1:**
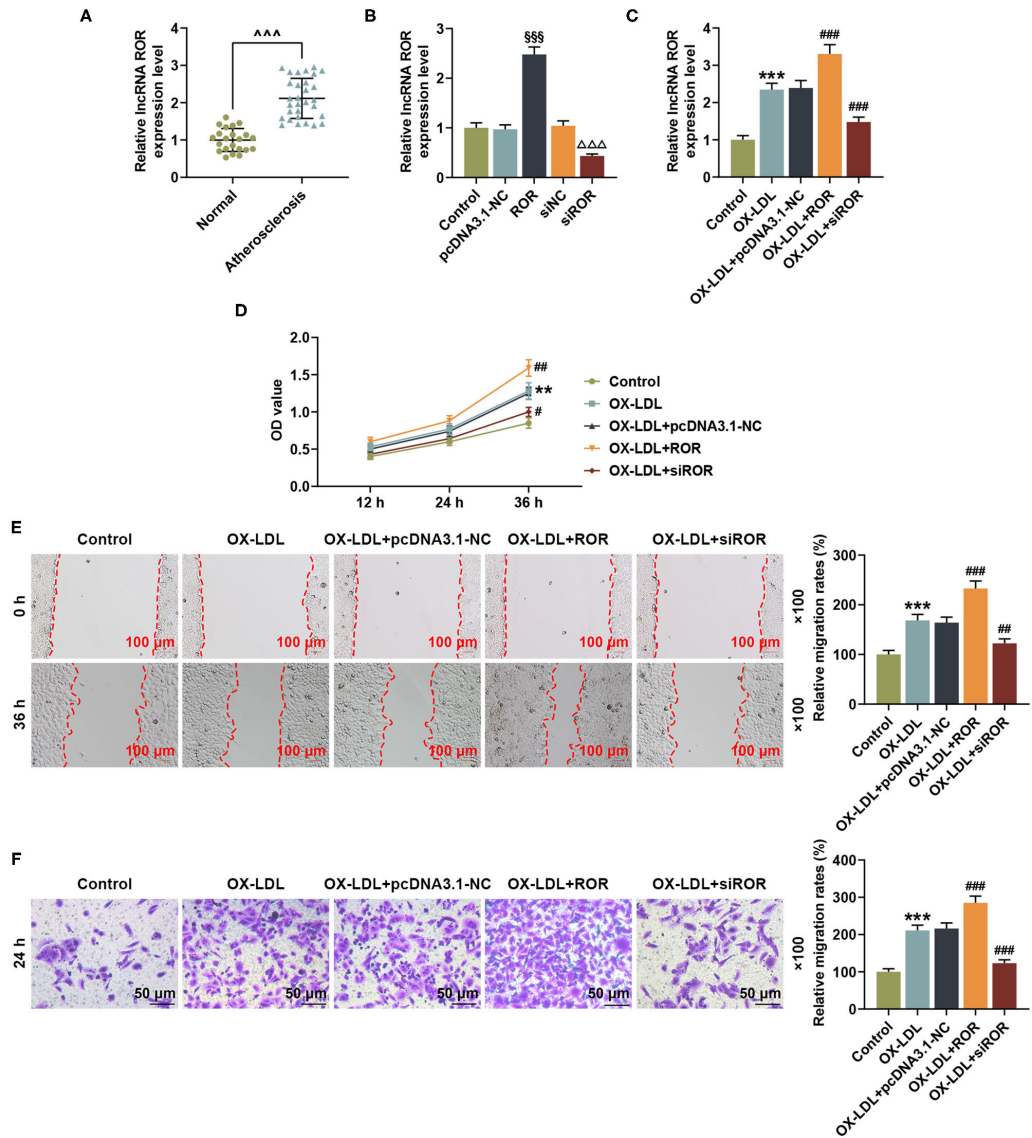
lncRNA ROR expression was upregulated in AS and ox-LDL-treated HUVECs and affected cell viability and migration. **(A)** The relative lncRNA ROR expression in peripheral venous blood from patients with AS (*n* = 30) and healthy donors (normal, *n* = 22) was measured with quantitative real-time polymerase chain reaction (qRT-PCR). β-Actin was used as an internal control. **(B)** Relative lncRNA ROR expression after the HUVECs were transfected with ROR overexpression plasmid, and siROR was measured by qRT-PCR. **(C)** Relative lncRNA ROR expression in the HUVECs after ox-LDL treatment and transfection of ROR overexpression plasmid, and siROR was measured with qRT-PCR. β-Actin was used as an internal control. **(D)** The viability of HUVECs after ox-LDL treatment and transfection of ROR overexpression plasmid and siROR was measured with CCK-8 assay at 12, 24, and 36 h. **(E)** The migration of HUVECs after ox-LDL treatment and the transfection of ROR overexpression plasmid and siROR at 0 and 36 h were measured with wound-healing assay. Magnification: × 100, scale bar = 100 μm. **(F)** The migration of HUVECs after ox-LDL treatment and transfection of ROR overexpression plasmid, and siROR at 24 h was measured with Transwell assay. Magnification: × 100, scale bar = 50 μm. All experiments have been performed independently in triplicate, and the data were expressed as mean ± standard deviation. Statistical significance was assessed by paired *t*-test and one-way ANOVA, followed by Tukey's *post-hoc* test. ^∧∧∧^*P* < 0.001 *vs*. normal; ^§§§^*P* < 0.001 *vs*. pcDNA3.1-NC; ^ΔΔΔ^*P* < 0.001 *vs*. siNC; **P < 0.01, ***P < 0.001 *vs*. control; ^#^*P* < 0.05, ^##^*P* < 0.01, ^###^*P* < 0.001 *vs*. ox-LDL+pc-DNA3.1-NC. ROR, regulator of reprogramming; AS, atherosclerosis; ox-LDL, oxidized low-density lipoprotein; HUVECs, human umbilical vein endothelial cells; siROR, small interfering RNA for lncRNA ROR; NC, negative control.

To examine the role of lncRNA ROR in AS, specimens of peripheral venous blood were obtained from AS patients (atherosclerosis, *n* = 30) and healthy donors (normal, *n* = 22). It can be noted from Figure 1A that the lncRNA ROR expression was upregulated in AS patients compared with that in healthy donors (Figure 1A, *P* < 0.001). In addition, the level of total cholesterol and triglyceride, respectively, in AS patients was higher than that in normal controls (Table 1).

### Overexpressed lncRNA ROR Increased Cell Viability and Migration of ox-LDL-Induced HUVECs, but lncRNA ROR Silencing Led to Opposite Results

The lncRNA ROR levels are abnormally expressed in AS patients, indicating that lncRNA ROR may be implicated in AS. To discover the effects of lncRNA ROR on ox-LDL-treated HUVECs, the HUVECs were pretreated with ox-LDL (19) and then transfected with lncRNA ROR overexpression plasmid and siROR. The transfection efficiency of lncRNA ROR was detected, which proved that lncRNA ROR expression was increased by lncRNA ROR overexpression but decreased by ROR silencing (Figure 1B, *P* < 0.001). As shown in Figure 1C, after ox-LDL treatment, the lncRNA ROR expression was upregulated (Figure 1C, *P* < 0.001), which enhanced the effects of ox-LDL on lncRNA ROR expression in the HUVECs, whereas lncRNA ROR silencing downregulated the lncRNA ROR expression (Figure 1C, *P* < 0.001). In accordance with Figure 1D, the viability of HUVECs was increased after ox-LDL treatment (Figure 1D, *P* < 0.01), and the upregulation of lncRNA ROR further enhanced the effects of ox-LDL on HUVEC viability, while ROR silencing led to a reduced HUVEC viability (Figure 1D, *P* < 0.05). Figures 1E,F demonstrated that, after ox-LDL treatment, the migration of HUVECs was increased (Figures 1E,F, *P* < 0.001). Meanwhile, the upregulation of lncRNA ROR further promoted the effects of ox-LDL on the migration of HUVECs, while ROR silencing decreased the migration of HUVECs (Figures 1E,F, *P* < 0.01).

### lncRNA ROR Could Bind With let-7b-5p Whose Expression Was Downregulated in AS

**Figure 2:**
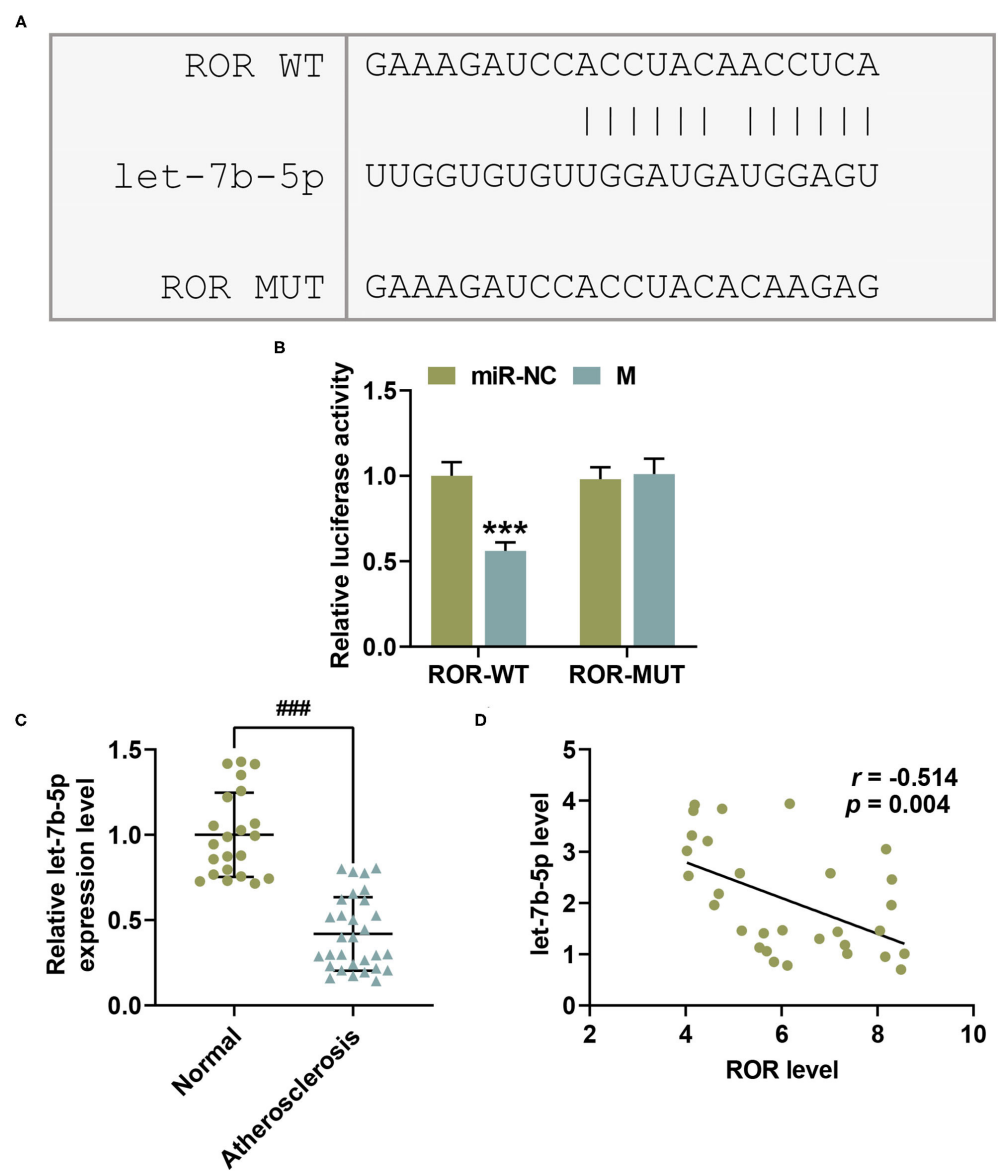
let-7b-5p inversely bound with lncRNA regulator of reprogramming (ROR), with its expression being downregulated in AS. **(A)** The predicted potential binding sites between lncRNA ROR and let-7b-5p are listed. **(B)** Dual-luciferase reporter assay confirmed that let-7b-5p could bind with lncRNA ROR. **(C)** The relative let-7b-5p expressions in peripheral venous blood from patients with atherosclerosis (*n* = 30) and healthy donors (normal, *n* = 22) were measured with qRT-PCR. U6 was used as an internal control. **(D)** Pearson's correlation test showed a negative correlation between let-7b-5p and lncRNA ROR. All the experiments have been performed independently in triplicate, and the data were expressed as mean ± standard deviation. Statistical significance was assessed by paired *t*-test and one-way ANOVA, followed by Tukey's *post-hoc* test. A correlation analysis of lncRNA ROR and let-7b-5p was performed using Pearson's correlation test. ***P < 0.001 *vs*. miR-NC; ^###^*P* < 0.001 *vs*. normal.

let-7b-5p was identified as the candidate miRNA binding to lncRNA ROR by RNA22 v2 microRNA target detection database. Their complementary binding sites are listed in Figure 2A. As shown in Figure 2B, compared with the miR-NC group, the luciferase activity was reduced by let-7b-5p mimic in the cells transfected with ROR-WT plasmid (*P* < 0.001), while no evident change was observed in the cells transfected with let-7b-5p mimic and ROR-MUT plasmid (Figure 2B).

In order to examine the role of let-7b-5p in AS, the let-7b-5p expression in specimens of peripheral venous blood from AS patients and healthy donors was measured. In the light of Figure 2C, let-7b-5p expression was decreased in AS patients (Figure 2C, *P* < 0.001). Moreover, a correlation analysis provided results indicating a negative correlation between the expressions of lncRNA ROR and let-7b-5p (Figure 2D, *r* = −0.514, *P* = 0.004).

### let-7b-5p Reversed the Effects of Upregulated lncRNA ROR on let-7b-5p Expression as Well as Cell Viability and Migration of the ox-LDL-Treated HUVECs

**Figure 3:**
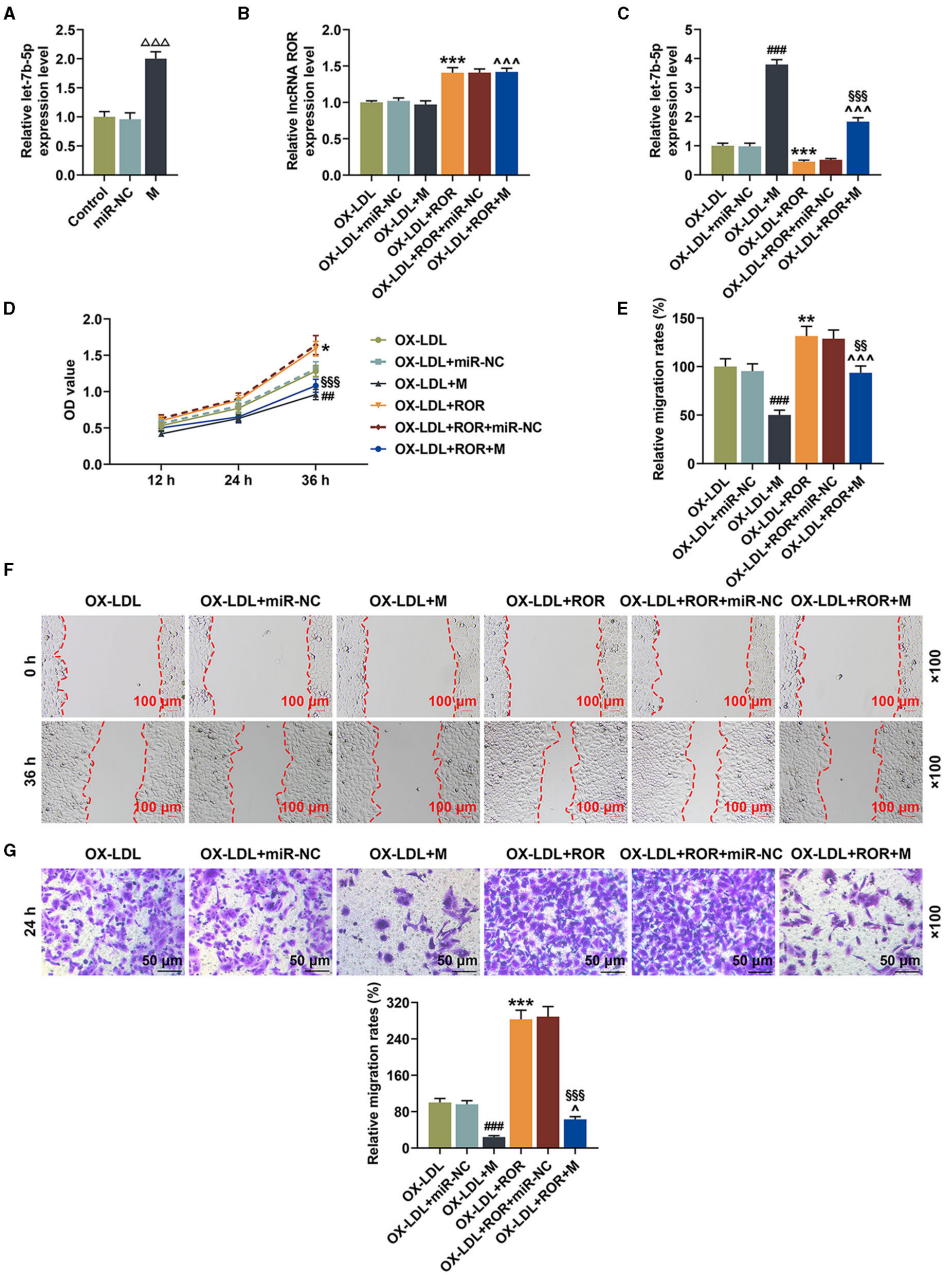
let-7b-5p reversed the effects of upregulated lncRNA regulator of reprogramming (ROR) expression on let-7b-5p expression as well as the cell viability and migration of ox-LDL-treated human umbilical vein endothelial cells (HUVECs). **(A)** The relative let-7b-5p expression after the HUVECs were transfected with let-7b-5p mimic was measured by qRT-PCR. **(B)** The relative lncRNA ROR in ox-LDL-treated HUVECs after transfection of ROR overexpression plasmid and let-7b-5p mimic was measured with qRT-PCR. **(C)** The relative let-7b-5p expression in ox-LDL-treated HUVECs after transfection of ROR overexpression plasmid and let-7b-5p mimic was measured with qRT-PCR. U6 was used as an internal control. **(D)** The viability of ox-LDL-treated HUVECs after transfection of ROR overexpression plasmid and let-7b-5p mimic was measured with CCK-8 assay at 12, 24, and 36 h. **(E,F)** The migration of ox-LDL-treated HUVECs after transfection of ROR overexpression plasmid and let-7b-5p mimic was measured with wound-healing assay at 0 and 36 h. Magnification: × 100, scale bar = 100 μm. **(G)** The migration of ox-LDL-treated HUVECs after transfection of ROR overexpression plasmid and let-7b-5p mimic was measured with Transwell assay at 24 h. Magnification: × 100x, scale bar = 50 μm. All experiments have been performed in triplicate, and data were expressed as mean ± standard deviation. Statistical significance was assessed by one-way ANOVA, followed by Tukey's *post-hoc* test. ^ΔΔΔ^*P* < 0.001 *vs*. miR-NC; ^##^*P* < 0.01, ^###^*P* < 0.001 *vs*. ox-LDL+miR-NC; *P < 0.05, **P < 0.01, ***P < 0.001 *vs*. ox-LDL; ^∧^*P* < 0.05, ^∧∧∧^*P* < 0.001 *vs*. ox-LDL+M; ^§§^*P* < 0.01, ^§§§^*P* < 0.001 *vs*. ox-LDL+ROR+miR-NC. miR-NC, microRNA—negative control.

To determine the possible role of let-7b-5p in ox-LDL-treated HUVECs, let-7b-5p mimic was transfected into the HUVECs treated by ox-LDL. As shown in Figure 3A, the expression of let-7b-5p was increased after the HUVECs were transfected with let-7b-5p mimic (Figure 3A, *P* < 0.001). As shown in Figure 3B, the expression of lncRNA ROR was increased after the ox-LDL-treated HUVECs were transfected with lncRNA ROR overexpression plasmids (Figure 3B, *P* < 0.001), while cells transfected with let-7b-5p mimic had no effect on the expression of lncRNA ROR. In addition, the increased let-7b-5p expression was also found in ox-LDL-treated HUVECs transfected with let-7b-5p mimic, while a decreased let-7b-5p expression was found in ox-LDL-treated HUVECs transfected with lncRNA ROR overexpression (Figure 3C, *P* < 0.001). In addition, let-7b-5p reversed the effect of lncRNA ROR overexpression on let-7b-5p expression in the ox-LDL-treated HUVECs (Figure 3C, *P* < 0.001).

In Figure 3D, it could be observed that the cell viability of the ox-LDL-treated HUVECs was decreased after the transfection of let-7b-5p mimic (Figure 3D, *P* < 0.01) but increased by upregulated lncRNA ROR expression, while let-7b-5p mimic partially reversed the effect of lncRNA ROR overexpression on the cell viability of ox-LDL-treated HUVECs (Figure 3D, *P* < 0.01). Figures 3E–G proved that the migration of ox-LDL-treated HUVECs was reduced after the transfection of let-7b-5p mimic (Figures 3E–G, *P* < 0.001). However, upregulated lncRNA ROR expression increased the migration of ox-LDL-treated HUVECs, and let-7b-5p reversed the effects of lncRNA ROR on increasing the cell migration of ox-LDL-treated HUVECs (Figures 3E–G, *P* < 0.05).

### let-7b-5p Reversed the Influence of Upregulated lncRNA ROR on Apoptosis-Related Protein Expressions

**Figure 4:**
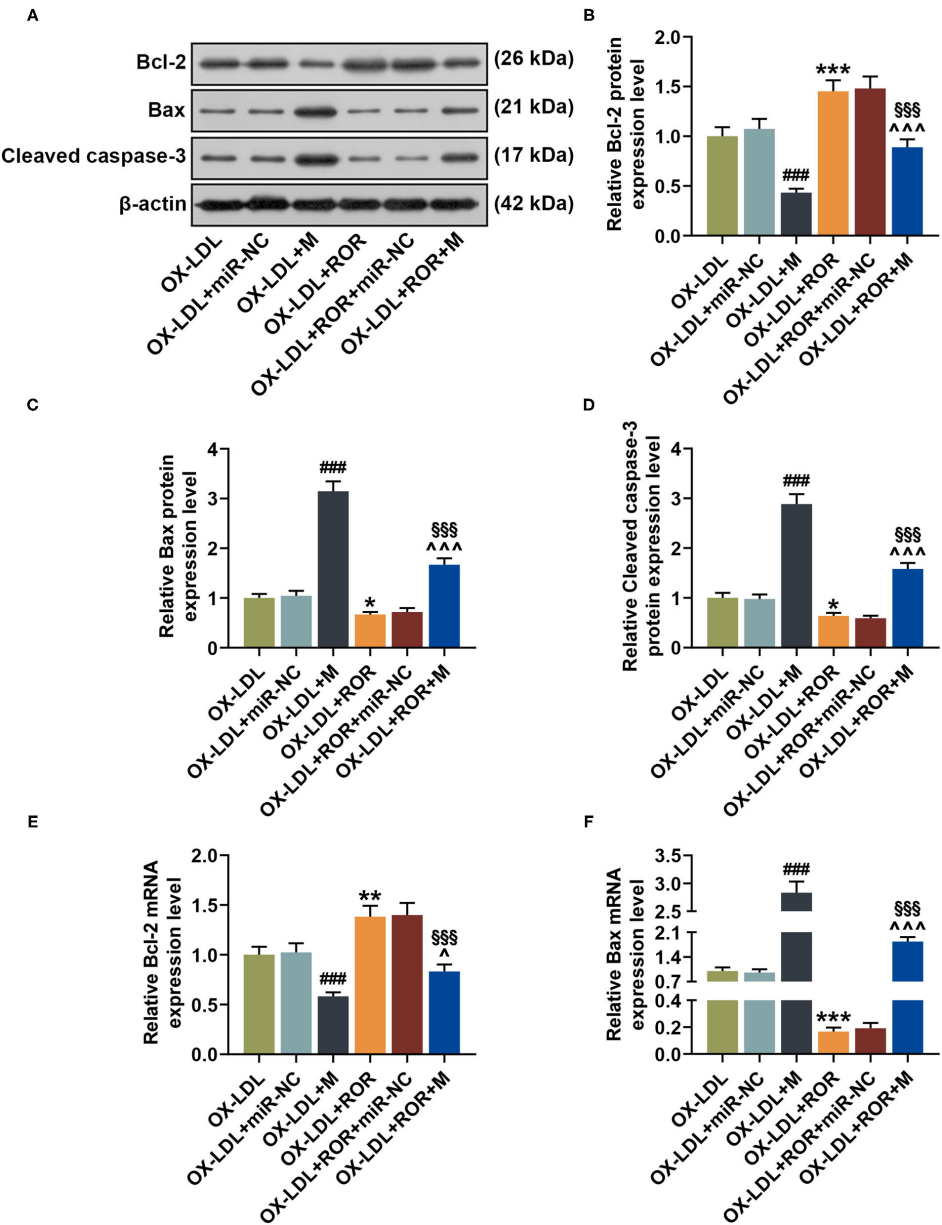
let-7b-5p reversed the effects of the upregulation of lncRNA regulator of reprogramming (ROR) on apoptosis-related protein expressions. **(A–D)** The relative protein/β-actin expressions of Bcl-2 **(B)**, Bax **(C)**, and cleaved caspase-3 **(D)** were measured by Western blot. β-Actin was used as an internal control. **(E,F)** The relative mRNA expressions of Bcl-2 **(E)** and Bax **(F)** were measured by qRT-PCR. β-Actin was used as an internal control. All experiments have been performed independently in triplicate, and data were expressed as mean ± standard deviation. Statistical significance was assessed by one-way ANOVA, followed by Tukey's *post-hoc* test. ^###^*P* < 0.001 *vs*. ox-LDL+miR-NC; *P < 0.05, **P < 0.01, ***P < 0.001 *vs*. ox-LDL; ^∧^*P* < 0.05, ^∧∧∧^*P* < 0.001 *vs*. ox-LDL+M; ^§§§^*P* < 0.001 *vs*. ox-LDL+ROR+miR-NC. Bcl-2, B-cell lymphoma-2; Bax, Bcl-2-associated X protein.

From Figures 4A–F, it could be observed that, after the transfection of let-7b-5p mimic, the expression of Bcl-2 was downregulated, while those of Bax and cleaved caspase-3 were upregulated (Figures 4A–F, *P* < 0.001). However, promoting lncRNA ROR expression upregulated Bcl-2 expression yet downregulated Bax and cleaved caspase-3 expressions (Figures 4A–F, *P* < 0.05). Additionally, let-7b-5p reversed the effects of upregulated lncRNA ROR expression on apoptosis-related protein expressions (Figures 4A–F, *P* < 0.05).

### HOXA1 Was the Target Gene of let-7b-5p and Its Expression Was Upregulated in AS

**Figure 5:**
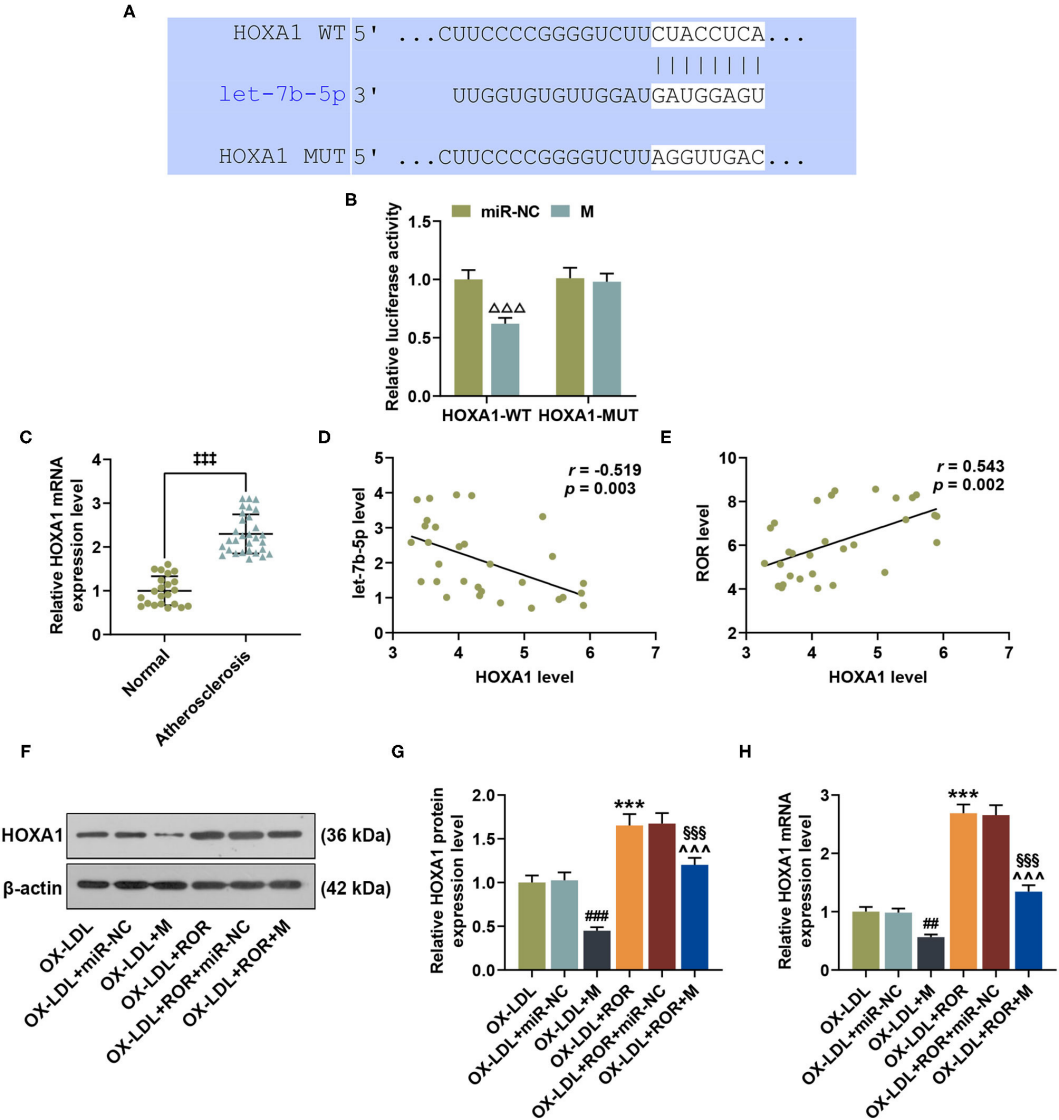
HOXA1 was the target gene of let-7b-5p, with its expression being upregulated in atherosclerosis (AS), and let-7b-5p reversed the effects of upregulated lncRNA regulator of reprogramming (ROR) on HOXA1 expression in ox-LDL-treated HUVECs. **(A)** The predicted potential binding sites between let-7b-5p and HOXA1 at wild-type 3′-UTR and mutated 3′-UTR of HOXA1 (HOXA1-WT and HOXA1-MUT) are shown. **(B)** Dual-luciferase reporter assay confirmed that HOXA1 was the target gene of let-7b-5p. **(C)** The relative HOXA1 expressions in peripheral venous blood from patients with AS (*n* = 30) and healthy donors (normal, *n* = 22) were measured with qRT-PCR. β-Actin was used as an internal control. **(D,E)** Pearson's correlation test detected a negative correlation between HOXA1 and let-7b-5p **(D)** and a positive correlation between HOXA1 and lncRNA ROR **(E)**. **(F,G)** The relative protein/β-actin expression of HOXA1 was measured by Western blot. β-Actin was used as an internal control. **(H)** The relative mRNA expression of HOXA1 was measured by qRT-PCR. β-Actin was used as an internal control. All experiments have been performed independently in triplicate, and data were expressed as mean ± standard deviation. Statistical significance was assessed by paired *t*-test and one-way ANOVA, followed by Tukey's *post-hoc* test. A correlation analysis of lncRNA ROR, let-7b-5p, and HOXA1 was performed using Pearson's correlation test. ^ΔΔΔ^*P* < 0.001 *vs*. miR-NC; ^‡*‡‡*^*P* < 0.001 *vs*. normal; ^##^*P* < 0.01, ^###^*P* < 0.001 *vs*. ox-LDL+miR-NC; ***P < 0.001 *vs*. ox-LDL; ^∧∧∧^ *P* < 0.001 *vs*. ox-LDL+M; ^§§§^*P* < 0.001 *vs*. ox-LDL+ROR +miR-NC. HOXA1, homeobox A1; 3′-UTR, 3′-untranslated regions.

On TargetScan, HOXA1 was identified as the target gene of let-7b-5p, whose complementary binding sites are listed in Figure 5A. As shown in Figure 5B, compared with the cells co-transfected with HOXA1-WT plasmid and miR-NC, the luciferase activity of the cells co-transfected with HOXA1-WT plasmid and let-7b-5p mimic was obviously inhibited (Figure 5B, *P* < 0.001), while no evident change was observed in the cells transfected with HOXA1-MUT plasmid and let-7b-5p mimic or control (miR-NC). The data above indicated that HOXA1 was the target gene of let-7b-5p. In addition, to explore the role of HOXA1 in AS, the expressions of HOXA1 in the peripheral venous blood from AS patients and healthy donors were detected, with the result manifesting that HOXA1 expression was upregulated in AS patients (Figure 5C, *P* < 0.001).

### Correlation Analysis of lncRNA ROR, let-7b-5p, and HOXA1

Pearson's correlation test was used to further detect the correlation of lncRNA ROR, let-7b-5p, and HOXA1. In line with Figures 5D,E, HOXA1 was negatively correlated with let-7b-5p (Figure 5D, *r* = −0.519, *P* = 0.003) but was positively correlated with lncRNA ROR (Figure 5E, *r* = 0.543, *P* = 0.002).

In addition, we found that HOXA1 expression was downregulated after the transfection of let-7b-5p mimic; however, upregulated lncRNA ROR expression promoted HOXA1 expression in ox-LDL-treated HUVECs (Figures 5F–H, *P* < 0.01). Furthermore, let-7b-5p reversed the effects of upregulated lncRNA ROR expression on HOXA1 expression in ox-LDL-treated HUVECs (Figures 5F–H, *P* < 0.01).

### The Roles and Functions of lnc ROR, let-7b-5p, and HOXA1 on HOXA1 Expression as Well as Cell Viability and Migration of the ox-LDL-Induced HUVECs

**Figure 6:**
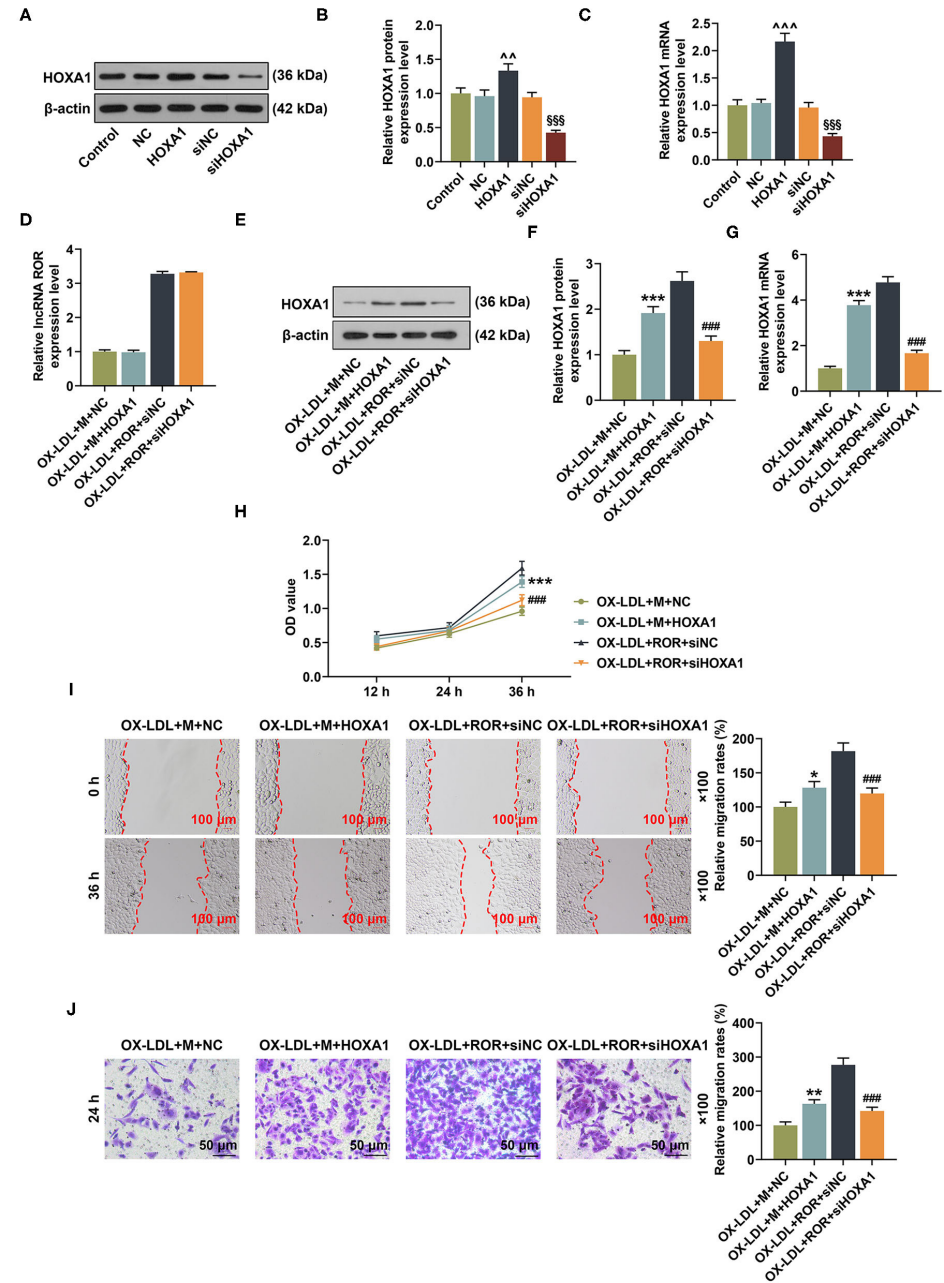
The roles and functions of lncRNA ROR, let-7b-5p, and HOXA1 on HOXA1 expression as well as cell viability and migration of ox-LDL-treated human umbilical vein endothelial cells (HUVECs). **(A–C)** After the HUVECs were transfected with HOXA1 overexpression plasmids or siHOXA1, the protein and mRNA expressions of HOXA1 were detected by Western blot and qRT-PCR. **(D)** The relative lncRNA regulator of reprogramming (ROR) in ox-LDL-treated HUVECs after transfection of let-7b-5p mimic and siHOXA1 was measured by qRT-PCR. **(E,F)** The relative HOXA1 protein/β-actin expression was determined using Western blot. β-Actin was used as an internal control. **(G)** The relative HOXA1 mRNA expression was measured by qRT-PCR. β-Actin was used as an internal control. **(H)** The viability of ox-LDL-treated HUVEC was measured with CCK-8 assay at 12, 24, and 36 h. **(I)** The migration of ox-LDL-treated HUVECs was measured with wound-healing assay at 0 and 36 h. Magnification: × 100, scale bar = 100 μm. **(J)** The migration rate of ox-LDL-treated HUVECs at 24 h was measured with Transwell assay. Magnification: × 100, scale bar = 50 μm. All experiments have been performed in triplicate, and data were expressed as mean ± standard deviation. Statistical significance was assessed by one-way ANOVA, followed by Tukey's *post-hoc* test. ^∧∧^*P* < 0.01, ^∧∧∧^*P* < 0.001 *vs*. NC; ^§§§^*P* < 0.001 *vs*. siNC; *P < 0.05, **P < 0.01, ***P < 0.001 *vs*. ox-LDL+M+NC; ^###^*P* < 0.001 *vs*. ox-LDL+ROR +siNC.

To further examine the roles and functions of lncRNA ROR, let-7b-5p, and HOXA1 in the ox-LDL-treated HUVECs, lncRNA ROR, and HOXA1 overexpression plasmids, let-7b-5p mimic and siHOXA1 were transfected into the cells. From Figures 6A–C, it could be concluded that the protein and mRNA expression of HOXA1 were increased after the HUVECs were transfected with HOXA1 overexpression plasmid but were decreased after the HUVECs were transfected with siHOXA1 (Figures 6A–C, *P* < 0.01). In addition, the effect of HOXA1 on lncRNA ROR expression was detected, and the result showed that there was no change of lncRNA ROR expression after the ox-LDL-treated HUVECs were transfected with HOXA1 overexpression plasmid (Figure 6D). HOXA1 overexpression plasmid transfection further upregulated HOXA1 expression in the ox-LDL+M+HOXA1 group as compared with that in the ox-LDL+M+NC group (Figures 6E–G, *P* < 0.001). However, after silencing HOXA1, HOXA1 expression was downregulated in the ox-LDL+ROR+siHOXA1 group in comparison with that in the ox-LDL+ROR+siNC group (Figures 6E–G, *P* < 0.001).

As demonstrated in Figures 6H–J, after the overexpression of HOXA1, the viability and migration of the ox-LDL-treated HUVECs in the ox-LDL+M+HOXA1 group were increased as compared with those in the ox-LDL+M+NC group (Figures 6H–J, *P* < 0.05). On the other hand, after silencing HOXA1 expression, the viability and migration of the ox-LDL-treated HUVECs in the ox-LDL+ROR+siHOX1 group were reduced in contrast with those in the ox-LDL+ROR+siNC group (Figures 6H–J, *P* < 0.001).

### The Effects of lncRNA ROR, let-7b-5p, and HOXA1 on Apoptosis-Associated Protein Expressions

**Figure 7:**
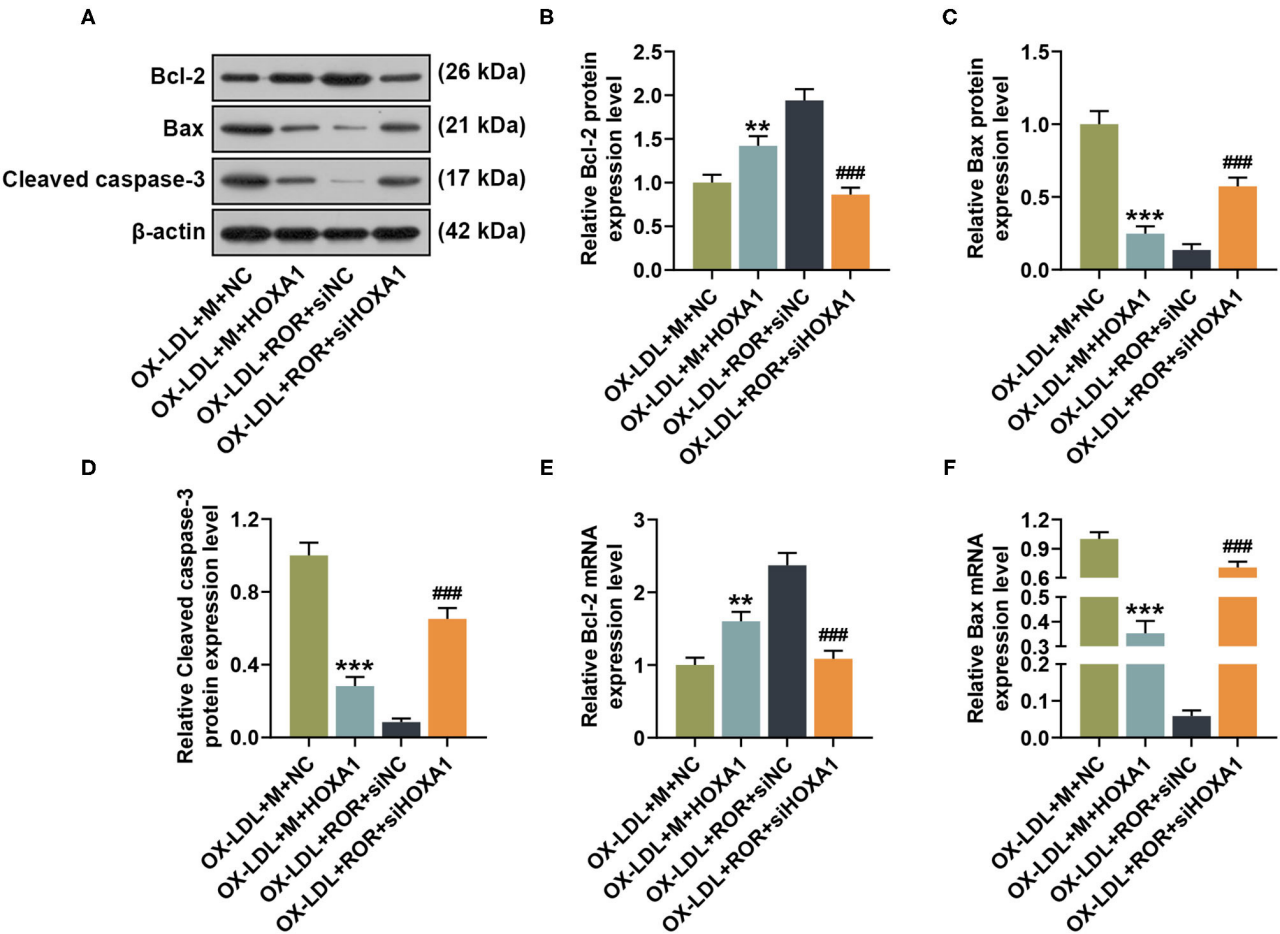
The roles and functions of lncRNA ROR, let-7b-5p, and HOXA1 on apoptosis-related protein expressions. **(A–D)** The relative protein/β-actin expression of Bcl-2 **(B)**, Bax **(C)**, and cleaved caspase-3 **(D)** was measured by Western blot. β-Actin was used as an internal control. **(E,F)** The relative mRNA expressions of Bcl-2 **(E)** and Bax **(F)** were measured by qRT-PCR. β-Actin was used as an internal control. All experiments have been performed independently in triplicate, and data were expressed as mean ± standard deviation. Statistical significance was assessed by one-way ANOVA, followed by Tukey's *post-hoc* test. **P < 0.01, ***P < 0.001 *vs*. ox-LDL+M+NC; ^###^*P* < 0.001 *vs*. ox-LDL+ROR+siNC.

In Figures 7A–F, after HOXA1 overexpression, in contrast with the ox-LDL+M+NC group, the expression of Bcl-2 was increased, while those of Bax and cleaved caspase-3 were decreased in the ox-LDL+M+HOXA1 group (Figures 7A–F, *P* < 0.01). However, after silencing HOXA1 expression, as compared with the ox-LDL+ROR+siNC group, we found that Bcl-2 expression was decreased but Bax and cleaved caspase-3 expressions were increased in the ox-LDL+ROR+siHOX1 group (Figures 7A–F, *P* < 0.001).

### The Effects of lncRNA ROR on the Viability, Migration, and Apoptosis-Associated Protein Expressions of HUVECs

**Figure 8:**
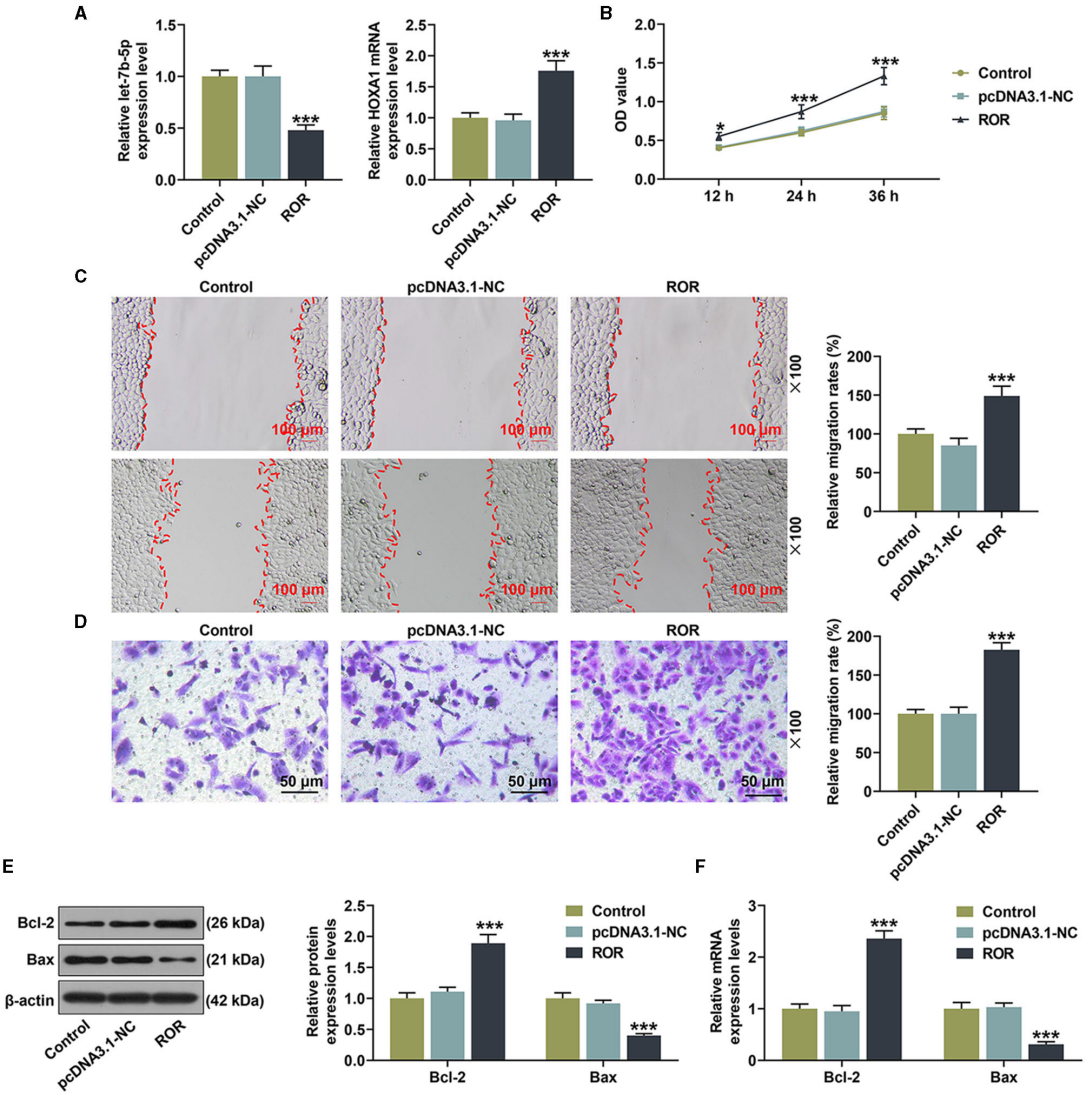
The roles and functions of lncRNA ROR on the viability, migration, and apoptosis-related protein expressions of human umbilical vein endothelial cells (HUVECs). **(A)** The mRNA expressions of let-7b-5p and HOXA1 were detected after the HUVECs were transfected with lncRNA ROR. **(B)** The viability was detected after the HUVECs were transfected with lncRNA ROR. **(C,D)** The migration was detected after the HUVECs were transfected with lncRNA ROR. **(E,F)** The relative protein/β-actin expressions of Bcl-2 and Bax were measured by Western blot. β-Actin was used as an internal control. ***P < 0.001 *vs*. pcDNA.3.1-NC.

All of the results mentioned above were performed under ox-LDL treatment, so we wanted to confirm if the effect of lncRNA ROR on the expression of let-7b-5p or HOXA1 and the cell viability and migration were dependent on this proatherogenic condition. As shown in Figure 8A, the effect of lncRNA ROR on the let-7b-5p and HOXA1 expressions was detected, and the result showed that lncRNA ROR inhibited the expression of let-7b-5p but promoted that of HOXA1 in the HUVECs (Figure 8A, *P* < 0.001). In addition, lncRNA ROR promoted the viability, and migration of the HUVECs (Figures 8B–D, *P* < 0.001). The expression of Bcl-2 was increased and that of Bax was decreased by lncRNA ROR (Figures 8E,F, *P* < 0.001). These results suggest that lncRNA ROR also affects the cell viability, migration, and apoptosis of HUVECs under normal conditions.

## Discussion

Ox-LDL plays a critical role in AS through acting on endothelial cells, smooth muscle cells, platelets, macrophages, and fibroblasts *via* LOX-1, which is a transmembrane glycoprotein functioning as a receptor for ox-LDL (19). A study indicated that ox-LDL could upregulate the expressions of multiple lncRNAs (24), but whether lncRNA ROR was also implicated in these diseases was incomprehensively understood. In our study, the ox-LDL-treated HUVECs upregulated lncRNA ROR expression, indicating that lncRNA ROR expression was promoted after ox-LDL treatment.

Previous studies uncovered the roles of lncRNAs in human cardiovascular diseases (25). Overexpressed lncRNA H19 in AS patients contributes to the progression of AS *via* activating the mitogen-activated protein kinase and nuclear factor-κB pathways [6]. Besides this, metastasis-associated lung adenocarcinoma transcript 1 is a lncRNA, and its activated Wnt/β-catenin signaling pathway is involved in the epithelial-to-mesenchymal transition of HUVECs, which has been found to exert a considerable influence on the onset of AS [7]. In addition, the expression of cancer susceptibility 11 (CASC11) is downregulated in AS, and overexpressed CASC11 suppresses the proliferation and promotes the apoptosis of vascular smooth muscle cells (26). In terms of lncRNA ROR, this has been reported to be involved in myocardial hypertrophy (27). In HASMCs, Hcy treatment was found to upregulate lncRNA ROR expression, which further promoted the proliferation and migration of HASMCs *via* the miR-195-5p/FGF2 axis. This mechanism might be possibly associated with AS pathophysiology (12). The downregulated lncRNA ROR inhibited the growth, migration, and angiogenesis of microvascular endothelial cells, possibly through the upregulation of miR-26 (28). In the current study, we also observed that, in AS, lncRNA ROR expression was upregulated, which promoted the biological behaviors of HUVECs, suggesting that upregulated lncRNA ROR expression could also promote the progression of AS.

Some proteins, such as Bax, Bcl-2, and caspase-3, play pivotal roles in cell apoptosis and are therefore considered as apoptosis-associated proteins (29). The upregulated lncRNA ROR expression could promote the expressions of Bax and cleaved caspase-3 and suppress Bcl-2 expression to promote the apoptosis of H/R-treated PC12 cells (30). In our study, however, we found that lncRNA ROR could further enhance the effect of ox-LDL on upregulating Bcl-2 expression and suppressing Bax and cleaved Caspase-3 expressions, demonstrating that upregulated lncRNA ROR expression could inhibit the apoptosis of HUVECs.

Recent studies investigated the roles of miRNAs and their interactions with lncRNAs in the pathogenesis of AS—for example, miR-185-5p interacts with retinal non-coding RNA3, which has a protective effect on AS (31). In addition, miR-21 could bind to taurine upregulated gene 1 (TUG1) and target the phosphatase and tensin homolog (PTEN), and the TUG1/miR-21/PTEN axis is closely related to AS progression (32). The lncRNA ROR can function as a molecular sponge to bind to relative miRNAs and affect their functions, downregulating the expression of targeted-miRs (8, 28). Fu et al. (33) pointed out that lnc-ROR functions as an endogenous microRNA sponge in pancreatic cancer cells, which regulates the expression of let-7 family gene, including let-7c-3p, let-7f-1-3p, let-7a-3p, let-7i-5p, and let-7f-3-3p. In our study, let-7b-5p was identified as the candidate miRNA binding with lncRNA ROR, which had a negative correlation with lncRNA ROR. Moreover, the upregulated let-7b-5p expression reversed the effects of overexpressed lncRNA ROR on cell viability and migration as well as the expression of apoptosis-related proteins. However, detailed molecular mechanisms remained to be determined.

HOXA1 is a HOX gene belonging to the HOX proteins that are a highly conserved family of transcription factors involved in gene regulatory networks in specification of anterior–posterior patterning (34). HOX genes have been found to be associated with oncogenesis, and HOXA1 is actively involved in several malignancies (35), such as endometrial cancer, cervical cancer, and non-small cell lung cancer (30, 36, 37). Resveratrol attenuated the expression of HOXA1 in human pulmonary artery endothelial cells and played a cardioprotective role (38). In AS, HOXA1 is a target gene of miR-99a-5p (39). In our study, HOXA1 was the target gene of let-7b-5p, which had a positive correlation with lncRNA ROR. A further analysis demonstrated that HOXA silencing reversed the effects of the upregulation of lncRNA ROR on the ox-LDL-treated HUVECs. The data suggested that the lncRNA ROR/let-7b-5p/HOXA1 axis may also be involved in AS development and progression.

It should be noted that there were some limitations in the current study. We only investigated the roles and effects of the lncRNA ROR/let-7b-5p/HOXA1 axis on the ox-LDL-treated HUVECs *in vitro*, but the results should be validated by *in vivo* experiments. In addition, apart from HOXA1, let-7b-5p may also play a role in AS by targeting other downstream targets, which needs further analysis.

In conclusion, we examined the role and effect of the lncRNA ROR/let-7b-5p/HOXA1 axis on ox-LDL-treated HUVECs by investigating the possible mechanisms of the lncRNA ROR/let-7b-5p/HOXA1 axis in the viability and migration of ox-LDL-induced HUVECs. The results of our study provide novel evidence for the role of lncRNA ROR and a potential therapeutic method for the treatment of AS.

## Data Availability Statement

The original contributions presented in the study are included in the article/Supplementary Material, further inquiries can be directed to the corresponding author/s.

## Ethics Statement

The studies involving human participants were reviewed and approved by The study was conducted after obtaining the approval from the Ethics Committee of Zhejiang Provincial People's Hospital (approval number: ZPPH2018020601). All the recruited patients have signed informed consents and agreed that their tissues would be used for clinical research. The patients/participants provided their written informed consent to participate in this study.

## Author Contributions

CY provided substantial contributions to the conception and design of this study and drafted the article or critically revised it for important intellectual content. BW, JJ, GY, CW, and FC performed data acquisition, data analysis, and interpretation. All authors gave final approval of the version to be published and agreed to be accountable for all aspects of the work in ensuring that questions related to the accuracy or integrity of the work are appropriately investigated and resolved.

## Funding

This work was supported by the Medical and Health Technology Project of Zhejiang Province in 2019 (grant no. 2019RC104).

## Conflict of Interest

The authors declare that the research was conducted in the absence of any commercial or financial relationships that could be construed as a potential conflict of interest.

## Publisher's Note

All claims expressed in this article are solely those of the authors and do not necessarily represent those of their affiliated organizations, or those of the publisher, the editors and the reviewers. Any product that may be evaluated in this article, or claim that may be made by its manufacturer, is not guaranteed or endorsed by the publisher.
