# Supplementary figures and images for: Overexpressed lncRNA ROR Promotes the Biological Characteristics of ox-LDL-Induced HUVECs via the let-7b-5p/HOXA1 Axis in Atherosclerosis
Source: Front Cardiovasc Med. 2021 Sep 13;8:659769. doi: 10.3389/fcvm.2021.659769 (PMC8473629; doi:10.3389/fcvm.2021.659769)

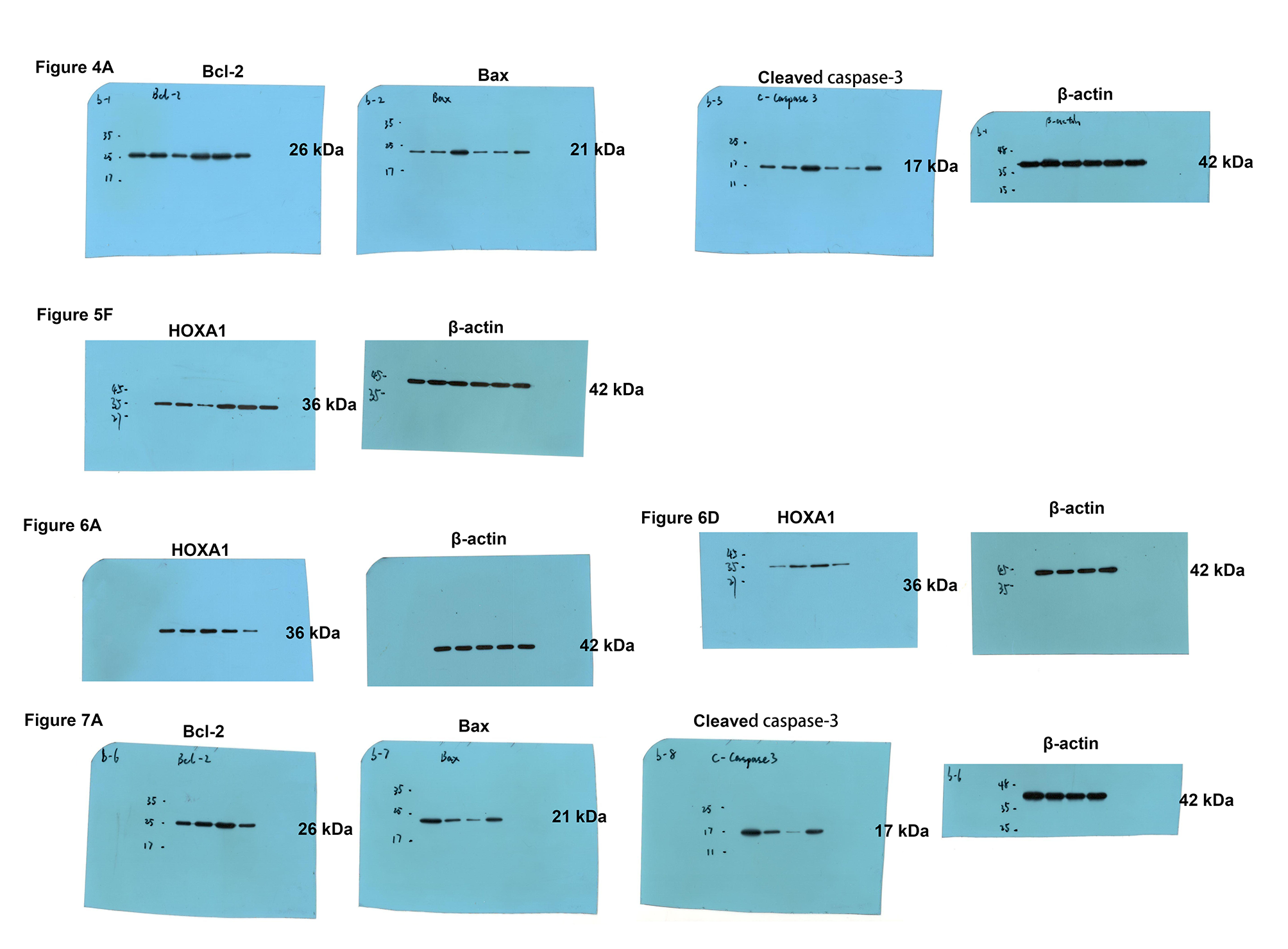

Supplement: Supplementary file 1 [file Image_1.TIF]

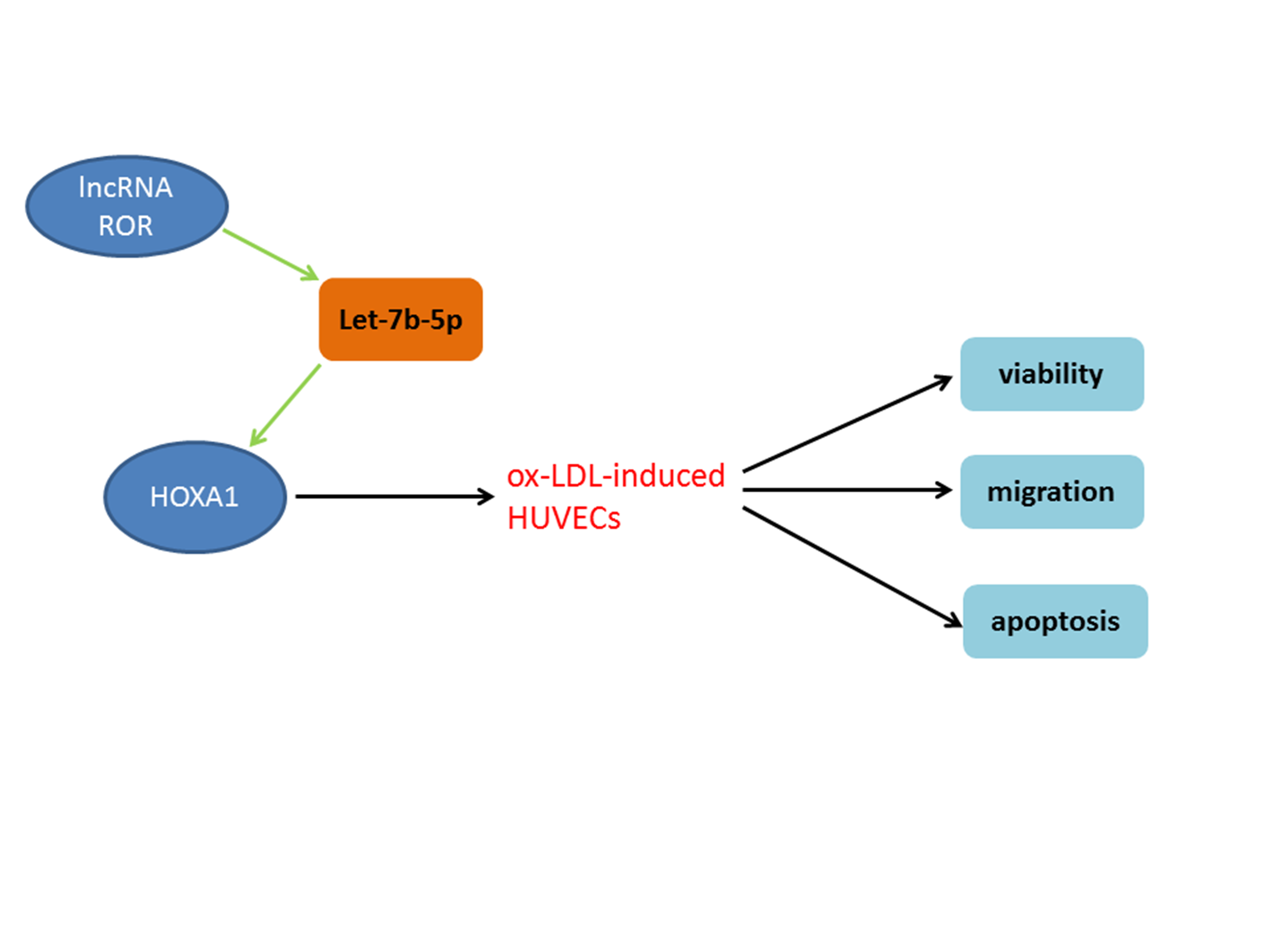

Supplement: Supplementary file 2 [file Image_2.TIF]

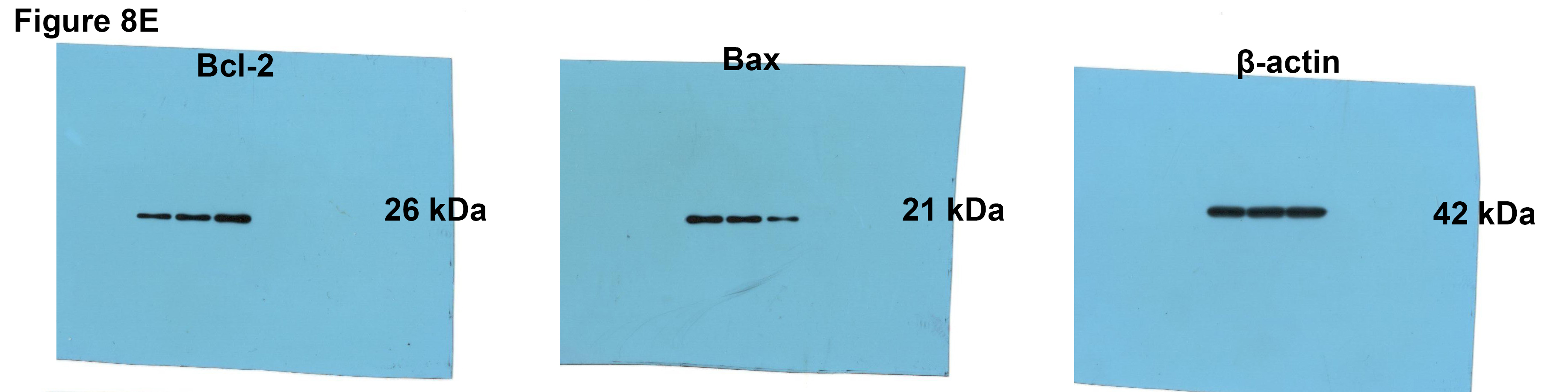

Supplement: Supplementary file 3 [file Image_3.TIF]
